# Use of NTRIP for Optimizing the Decoding Algorithm for Real-Time Data Streams

**DOI:** 10.3390/s141018878

**Published:** 2014-10-10

**Authors:** Zhanke He, Wenda Tang, Xuhai Yang, Liming Wang, Jihua Liu

**Affiliations:** 1 National Time Service Center, The Chinese Academy of Sciences, Xi'an 710600, China; E-Mails: yyang@ntsc.ac.cn (X.Y.); liujh@126.com (J.L.); 2 University of the Chinese Academy of Sciences, Beijing 100049, China; 3 Key Laboratory for Precision Navigation and Timing Technology, National Time Service Center, Chinese Academy of Sciences, Xi'an 710600, China; 4 Xidian University, Xi'an 710071, China; E-Mail: wdtang@stu.xidian.edu.cn (W.T.); wanglm@mail.xidian.edu.cn (L.W.)

**Keywords:** NTRIP, real-time, BeiDou, GNSS, international GNSS Monitoring and Assessment System, RTCM

## Abstract

As a network transmission protocol, Networked Transport of RTCM via Internet Protocol (NTRIP) is widely used in GPS and Global Orbiting Navigational Satellite System (GLONASS) Augmentation systems, such as Continuous Operational Reference System (CORS), Wide Area Augmentation System (WAAS) and Satellite Based Augmentation Systems (SBAS). With the deployment of BeiDou Navigation Satellite system (BDS) to serve the Asia-Pacific region, there are increasing needs for ground monitoring of the BeiDou Navigation Satellite system and the development of the high-precision real-time BeiDou products. This paper aims to optimize the decoding algorithm of NTRIP Client data streams and the user authentication strategies of the NTRIP Caster based on NTRIP. The proposed method greatly enhances the handling efficiency and significantly reduces the data transmission delay compared with the Federal Agency for Cartography and Geodesy (BKG) NTRIP. Meanwhile, a transcoding method is proposed to facilitate the data transformation from the BINary EXchange (BINEX) format to the RTCM format. The transformation scheme thus solves the problem of handing real-time data streams from Trimble receivers in the BeiDou Navigation Satellite System indigenously developed by China.

## 1. Introduction

“Networked Transport of RTCM via Internet Protocol” (NTRIP) stands for an open application-level protocol developed by the Federal Agency for Cartography and Geodesy of Germany (BKG) and certificated by RTCM. It has become a generic and stateless protocol based on the Hypertext Transfer Protocol HTTP/1.1 [1]. With the earliest version of NTRIP similar to the Internet Radio system, its version 1.0 was released in September 2004. Its Version 2.0, published in 2011, is widely used. The NTRIP has become a RTCM standard designed for disseminating differential correction data or other kinds of streaming data from Global Navigation Satellite System (GNSS) to stationary or mobile users over the Internet [2].

**Figure 1.**
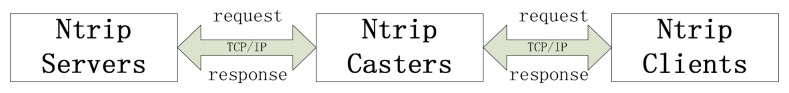
Framework of NTRIP.

The NTRIP is implemented in three system software components: NTRIP Clients, NTRIP Servers and NTRIP Casters. The NTRIP Caster is the actual HTTP server program whereas NTRIP Client and NTRIP Server act as HTTP clients. The relationship between these three components is illustrated in Figure 1.

The NTRIP Server is responsible for the transmission of real-time data streams from a reference station to a NTRIP Caster. The NTRIP Caster acts as a HTTP server which receives streaming RTCM data from one or more NTRIP Servers and in turn streams the RTCM data to one or more NTRIP Clients via the internet. The NTRIP Caster is designed to manage the web access of the NTRIP Servers and NTRIP Clients. The management is implemented in terms of mount points, port number, password and username, *etc.*, set for reference stations. The NTRIP Client receives streaming RTCM data from the NTRIP Caster to apply as real-time corrections to a roving GNSS receiver.

The NTRIP has been widely used in the Continuous Operational Reference System (CORS), Wide Area Augmentation System (WAAS) and the Satellite Based Augmentation Systems (SBAS). As the BeiDou Navigation Satellite System (BDS) indigenously developed by China contributes to navigation and positioning services for the Asian-Pacific region, the construction of the international GNSS Monitoring and Assessment System (iGMAS) is accelerating [3]. The type of application protocols is the critical factor for tracking network constructors to determine. This paper aims to optimize the decoding algorithm of NTRIP Client data streams and the user authentication strategy of the NTRIP Caster. The proposed method greatly enhances the handling efficiency and significantly reduces the data transmission delay compared with BKG NTRIP. Meanwhile, a transcoding method is proposed to facilitate the data transformation from the BINEX format to the RTCM format. The transformation scheme thus solves the problem of handing real-time data streams from multi receivers in the BeiDou Navigation Satellite System indigenously developed by China.

## 2. Disadvantages of BKG NTRIP Software

### 2.1. Unable to Deal with BeiDou Data Streams of iGMAS

The iGMAS provides the BeiDou data format according to RTCM 1040 3.1 and makes further extensions such as the frame structure, type of information summary, data type, data field and message. For the data type defined in RTCM 3.1, the type numbers of the observed data and ephemeris data for BeiDou are 1104 and 4011, respectively. The BKG software is so powerful that it is supposed to be able to deal with the real-time data streams of BeiDou navigation satellites, as mentioned in the BKG Ntrip Client (BNC) manual. However, after the official announcement of the Interface Control Document (ICD) 2.0 in December 2013, no agreement has been made between the BeiDou and RTCM. BNC is unable to deal with BeiDou data streams of iGMAS.

### 2.2. Long Processing Time

**Figure 2.**
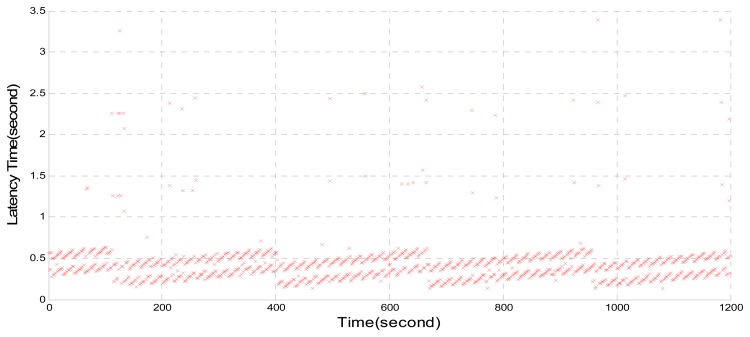
Time delay by using Trimble NetR9, BKG Caster 0.15 and BNC 2.10.

The data processing from a NTRIP Server to a BKG NTRIP Caster and decoding in BNC causes a great time delay. Figure 2 shows the time delay by using the Trimble NetR9 as a NTRIP Server to send data streams, BKG Caster 0.15 and BNC 2.10 for decoding. It can be seen that there are quite a few delays of longer than one second in the Local Area Network, with a maximum delay of 3.4 s.

## 3. Optimization of NTRIP

### 3.1. Decoding in the NTRIP Client

Since the BNC is unable to decode the BeiDou data of iGMAS, a decoding program for the NTRIP Client is developed independently by the authors. The program aims to decode RTCM3.1 data of GPS, GLONASS and BeiDou.

Some arithmetic factors are not predetermined in the BNC. These factors are calculated repeatedly while decoding, which greatly affects the decoding efficiency. The impact is even worse in processing massive multi-GNSS data. For the development of the NTRIP Client, the pre-calculation of arithmetic factors and applications of macro definitions were employed to reduce the time delay due to the function jump. This can reduce the RTCM decoding time to a certain extent.

**Figure 3.**
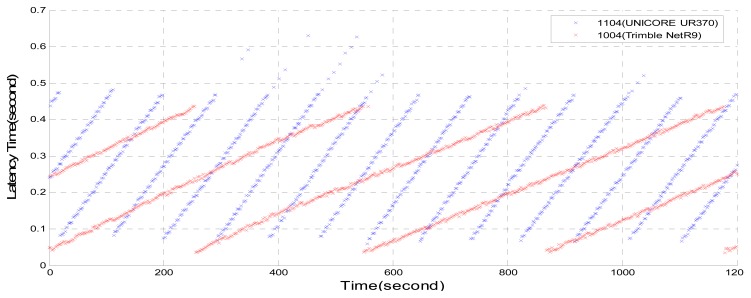
Time delay by using UNICORE UR370, Trimble NetR9, BKG Caster and proposed NTRIP Client.

From Figure 3, it can be seen that the proposed NTRIP Client leads to a reduction in the delay. However, in the area network there still exists 0.6 s delay. It is suggested that the delay results from the BKG NTRIP Caster.

### 3.2. NTRIP Caster

**Figure 4.**
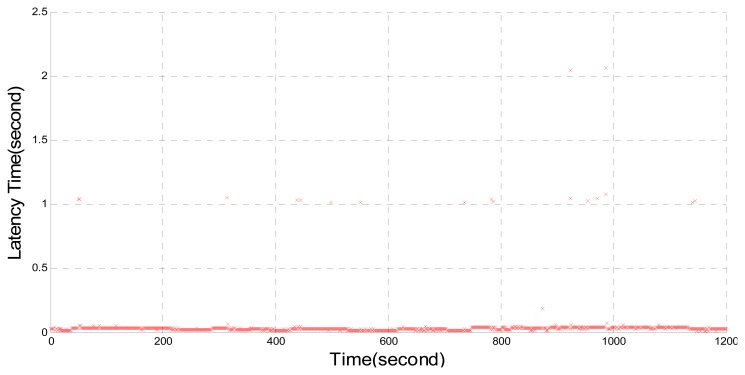
Time delay by using Trimble NetR9, proposed NTRIP Caster and BNC.

According to the protocol information, a new NTRIP Caster is independently developed by the authors and is used in combination with BKG NTRIP Client. Figure 4 shows the delay caused by the developed NTRIP Caster where delays of 1–2 s can still be found. Compared with the delay conditions in Figures 2 and 3, it can be seen that the self-developed NTRIP Caster reduces the delay but the effect is limited. Therefore, it is suggested that both the BKG NTRIP Caster and NTRIP Client might be problematic.

The BKG NTRIP Caster uses advanced data structures such as AVL trees [4] and mutex locks [5] for data streams. However, it is found that these designs are inconsistent with the nature of NTRIP Caster. Firstly, the balanced binary tree in the NTRIP Caster is useless to shorten the data searching duration (even if no data searching is needed). It just needs a hash table [6] to store the user account. After all, the key performances of NTRIP Caster are the reduction of the data transfer delay and quick safety authentication. Secondly, no modification on the data stream is needed in the NTRIP Caster. Therefore, no lock is needed for the data transfer. The data transfer in the critical section [7] is the atomic operation [8] of operating systems. Frequent access to the critical section will keeps the applications in a kernel mode [7] which is time-consuming and goes against the nature of NTRIP Caster.

**Figure 5.**
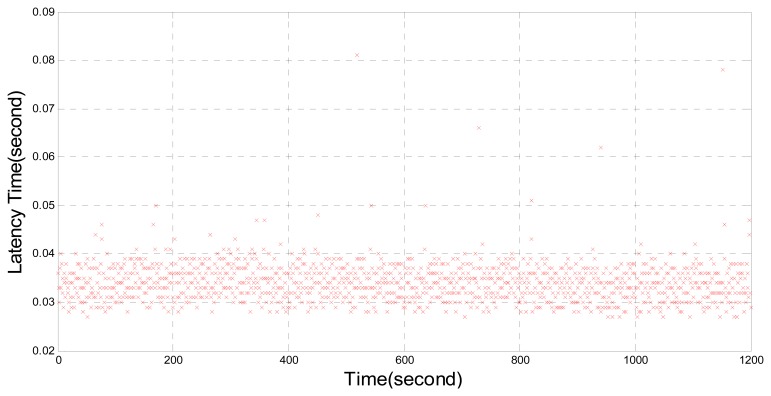
Time delay by using UNICORE UR370, Trimble NetR9, proposed NTRIP Client and NTRIP Caster.

To solve these problems, optimizations have been made by the authors on the NTRIP Caster in this paper. For NTRIP Caster, the Observer Design Pattern [9] was used. The NTRIP Client subscribed to the mount point data stream from the NTRIP Caster by invoking multi-station socket connections. After the identification by the NTRIP Server, the NTRIP Caster was connected and the data was sent to the NTRIP Client. A hash table was applied in the user identification process to speed up the matching of user names and passwords. In this way, the time delay during the data transfer in NTRIP Caster is significantly reduced. This strategy for data transmission based on the developed NTRIP Caster and NTRIP Client has better effect on the prevention of time delay in the area network by confining the average delay within 0.02–0.04 s, as shown in Figure 5.

## 4. Transformation from BINEX to RTCM

**Figure 6.**
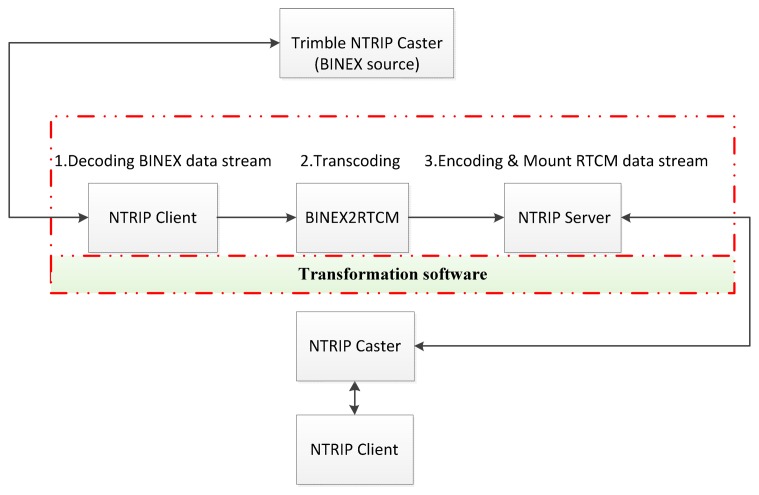
Transformation process from BINEX to RTCM.

There are many types of receivers. The Trimble NetR9 is able to output the BeiDou observed and ephemeris data in the BINEX format. However, it is not available for data in the RTCM format. To this end, the format of real-time data streams is unified in this study by transforming the BINEX format output by Trimble NetR9 to RTCM format. The BINEX, *i.e.*, abbreviation of BINary EXchange, is the standard binary format used for research and business in the GNSS. The BINEX format can encapsulate universal ASCII exchange format such as RINEX, IONEX, SP3 SINEX, *etc.* It also includes corresponding data and metadata [10]. The transformation process from BINEX to RTCM is depicted in Figure 6.

**Figure 7.**
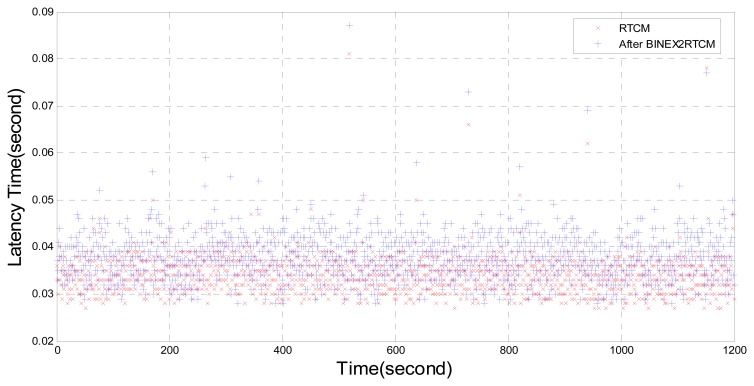
Time delay by using Trimble NetR9, proposed NTRIP Client, NTRIP Caster and BINEX2RTCM.

The transformation method of the RTCM format from the BINEX format needs additional transcoding work and makes the delay a bit longer than that without the transcoding (Figure 7). However, the overall delay is acceptable.

The developed algorithm and software have been used in the iGMAS data center in Xi'an. It provides real-time data streams for the data centre and other iGMAS data analysis centers for PANDA software to conduct precision positioning [11] and precision clock difference research.

## 5. Conclusions

In order to facilitate the application of data stream transmission in the BeiDou Navigation System, this paper has proposed an optimized NTRIP system consisting of NTRIP Server, NTRIP Caster and NTRIP Client for real-time data transmission. The proposed NTRIP reduced the delay in the data transmission and enabled the data handing for Trimble receivers.
